# The ethics of explantation

**DOI:** 10.1186/s12910-021-00690-8

**Published:** 2021-09-08

**Authors:** Sven Ove Hansson

**Affiliations:** Department of Learning, Informatics, Management and Ethics, Karolinska Institutet, 171 77 Stockholm, Sweden

**Keywords:** Battery, Breast implant, Cochlear implant, Explantation, Implant, Kant, Neurostimulator, Ownership, Pacemaker, Supplemented body part, Ventricular assistive device

## Abstract

**Background:**

With the increased use of implanted medical devices follows a large number of explantations. Implants are removed for a wide range of reasons, including manufacturing defects, recovery making the device unnecessary, battery depletion, availability of new and better models, and patients asking for a removal. Explantation gives rise to a wide range of ethical issues, but the discussion of these problems is scattered over many clinical disciplines.

**Methods:**

Information from multiple clinical disciplines was synthesized and analysed in order to provide a comprehensive approach to the ethical issues involved in the explantation of medical implants.

**Results:**

Discussions and recommendations are offered on pre-implantation information about a possible future explantation, risk–benefit assessments of explantation, elective explantations demanded by the patient, explantation of implants inserted for a clinical trial, patient registers, quality assurance, routines for investigating explanted implants, and demands on manufacturers to prioritize increased service time in battery-driven implants and to market fewer but more thoroughly tested models of implants.

**Conclusion:**

Special emphasis is given to the issue of control or ownership over implants, which underlies many of the ethical problems concerning explantation. It is proposed that just like transplants, implants that fulfil functions normally carried out by biological organs should be counted as supplemented body parts. This means that the patient has a strong and inalienable right to the implant, but upon explantation it loses that status.

## Background

As medical technology develops, more and more types of medical devices are implanted into the bodies of patients. With the increased prevalence of implants follows a large number of explantations. Several types of implants have had to be explanted due to manufacturing defects [1, 2]. Implants are also removed for a wide range of other reasons, including recovery making them unnecessary, battery depletion, availability of new and better models, and patients wishing to get rid of them. Explantation gives rise to a wide range of ethical issues, but the discussion of these problems is scattered over many clinical disciplines. This article provides a comprehensive discussion of the ethics of explantation, aiming to unify discussions in various clinical disciplines.

## Methods

This contribution is based on an extensive explorative literature search. The primary search was performed in PubMed in July 2020. The following combinations of search terms were used:

- explant + ethics
- explantation + ethics
- implant + remove + ethics
- implant + removal + ethics
- implant + failure + ethics
- implant + replacement + ethics

No limitations on publication years or language were applied. Judging by abstracts and keywords, no important literature was lost due to the author’s linguistic limitation (English, German, French, Spanish, Swedish, Danish and Norwegian). Around 1300 texts were found. Searches were also made on Philosopher’s Index and PhilPapers with the same search terms, but this did not add any literature not found in PubMed. Based on abstracts, keywords and, when needed and available, online full texts, literature containing no or obviously redundant information on the ethics of explantation was excluded. This resulted in a selection of about 110 articles that were read in detail. Further ad hoc explorative searches were made in PubMed for clinical and technical information on some of the identified issues, in particular patient registers, implant failures, battery time, and the performance of the medical device industry. Finally, the ethical issues were analysed with particular attention to comparison between approaches to explantation in different clinical disciplines.

## Results

### Risk assessments of explantations

Decisions on whether or not to explant require that the positive and negative effects of the available options are weighed against each other. If explantation is risky, it may be better to leave the implant in the body even if it does not function as desired or is for some reason no longer needed. Even if an implant has been recalled by the manufacturer, it is not self-evident that it should be removed. If explantation (with or without replacement) is a risky procedure, then it may be preferable to leave the recalled device in place [3]. After the Bjork-Shiley convexo-concave heartvalve was recalled in 1986, difficult decisions had to be made on which patients should have the implant explanted and replaced. Unfortunately, both options were connected with risks, which had to be weighed against each other [4]. A similar problem can arise for old pacemaker leads. Both alternatives—abandoning or removing the leads—can give rise to complications, and a decision on whether or not to explant the leads must therefore be based on an individual assessment of the risks associated with each of the options [5]. If an implant can put the patient at risk in magnetic resonance imaging (MRI), then that speaks in favour of explanting the implant when it is no longer needed [6, 7]. It is important that the risk assessments of explantations are wide enough, and not limited to immediate effects at the site of surgery. For instance, in decisions whether breast implants with manufacturing defects should be explanted, the risk of rupture or leakage from a retained implant may have to be weighed against negative psychological effects on the patient’s body image [8, 9].

When new and better implants become available, it can in some cases seem reasonable to explant and replace an old implant although it is still functioning. The potential advantages of a successful replacement will then have to be weighed against the risks of the intervention. These include the risk that the new device will not work satisfactorily, so that the operation results in worse rather than improved functionality. When this situation arises for patients with old cochlear implants in both ears, the risk can be mitigated by making the replacement in only one of the ears [3]. For implants that do not have a duplicate, the decision may be more difficult.

Some implants are intended only for temporary use, and may have to be explanted to avoid complications. This applies for instance to ureteral stents, for which complications can ensue if they are kept longer than the recommended indwelling time. Routines are needed to ensure that ureteral stents are not forgotten, and patients who do not keep appointments for removal will have to be contacted for a new appointment [10]. Similarly, orthopaedic implants that guide bone growth in children have to be explanted when they are no longer needed [11]. In some cases it may be an open question at the time of implantation whether the implant will be temporary or not. One example is LVADs (left ventricle assist devices), which have traditionally been used as bridge devices in patients waiting for a heart transplantation. In some patients an LVAD can function as a bridge to recovery, which means that the LVAD can be explanted when it is no longer needed [12].

In a letter to a plastic surgery journal in 1992, a surgeon criticized colleagues for unnecessarily removing breast implants due to “unfounded rumours” of a risk of immunological or malignant disease. He maintained that “[s]ome leaders of organized plastic surgery” condoned medically unjustified explantations as a means to “placate the legal profession and protect ourselves from litigation” [13]. If true, this would be ethically highly problematic, since it is hardly possible to defend that a medical intervention that does not benefit the patient is performed in order to benefit the physician.

### The responsibility of manufacturers

Manufacturers of implants have a considerable responsibility for reducing the need for explantations. Improvement is needed in two major areas, namely service time and quality control.

In practice, the major issue concerning service time is battery depletion. More and more patients receive battery-driven implants such as pacemakers, cardioverter defibrillators, ventricular assist devices, and a wide variety of neurostimulators, chemical sensors, and drug delivery systems. Batteries are an increasing portion of the volume of implants, since the other components have become smaller [14]. Implant replacement or battery exchange usually entails invasive surgery and therefore also a risk of infection. This makes it important to prolong the service time of implanted electrical devices [15]. There are several ways to achieve this. Batteries can be improved, and devices can be made more energy efficient. Furthermore, several methods have been proposed for wireless power transfer from outside the body to medical implants. Potential means of power transfer include electromagnetic induction, radio waves, and ultrasound waves [14]. An enzymatic glucose/oxygen fuel cell that draws its fuel from the blood stream has also been proposed [16].

However, the current economic incentives structure impedes the development of devices with a longer lifetime. Implants that last longer, thereby reducing the risks associated with explantation and replacement, could reduce the profits of manufacturers and profit-driven healthcare providers. This may be the reason why pacemakers are not built to last as long as would be technologically possible [15, 17]. The major manufacturers put new pacemaker models on the market about every 6 or 12 months, often with new, not very urgent functionalities that draw upon the battery [18, 19]. It has been proposed that pacemakers should have “only the pacemaker generator features that have proven clinical benefit” in order to increase their service time [17]. This would seem to require a change in the incentives for development and marketing of these devices.

The other major responsibility of manufacturers is to minimize the number of implants that have to be explanted and (usually) replaced due to device failure. In order to achieve this, the quality assurance system for implants has to be improved. What is needed is essentially the same process as for pharmaceutical drugs, namely the combination of (1) a series of pre-market clinical trials on a limited number of patients, showing that the device actually works and does not have frequent serious side effects, and (2) continued post-market studies in order to discover problems that were not observed in pre-market trials, for instance due to rarity, patient selection, or the limited time of observation. Full transparency of these studies is needed to ensure that independent researchers can analyze the data and compare the patient benefit of different products. But unfortunately, these procedures are still much less developed for implanted devices than for drugs. This applies not least to the European Union, whose regulatory system delegates approvals to pro-profit certification firms and keeps injury and malfunction reports secret with the justification that they are commercially sensitive for manufacturers [1].

In 1993, a group of orthopaedic surgeons complained that manufacturers “scarcely let a year go by without introducing a ‘new improved’ joint replacement which offers hitherto undreamt of (and unproved) advantages over the older designs”. Some of these are later “quietly withdrawn from the market” when they have proven to be substantially inferior to previously available models. The authors called this a “fashion trade” that “is causing patients unnecessary pain and distress” through early failures that lead to explantation and replacement of the defective implants [20]. The “fashion trade” still persists because the economic incentives still encourage it. Fewer but more thoroughly tested models of joint replacements would have increased patient safety. The situation is similar for other implants, not least pacemakers, which also have a short production life-cycle due to frequent changes [21]. Just as for follow-up drugs (“me-too drugs”), it is important for regulators to distinguish between products that contribute to incremental improvement and products that offer no demonstrable advantages over already available models [22].

The need for registers of patients with implants became acute after the Poly Implant Prothèse (PIP) breast implant scandal in 2010. Due to the lack of such registers, it was difficult to collect epidemiological data to determine what risks these implants were associated with. It was also difficult and sometimes impossible to identify affected women in order to offer them medical check-ups and, if needed, explantation. In several countries this experience led to the introduction of registers of patients with breast implants [2, 23]. There is a similar need of registers for other implants, such as artificial joints [20], growth rods for childhood scoliosis [24], and intraocular lenses [25].

### Elective explantations

Some patients have required explantation of an implant despite being told that this would be detrimental to their health and perhaps even life-threatening. Such cases appear to be rare, but they tend to be difficult to deal with ethically. They involve a conflict between two basic principles of medical ethics. From the viewpoint of patient autonomy, a patient with decisional capacity should be able to decline or discontinue a treatment. If only this principle is taken into consideration, then it can be argued that a patient always has the right to have an implant removed, whatever the consequences. However, according to the principle of non-maleficence, physicians should not perform harmful interventions on patients. If a patient asks for a medically harmful explantation, then the two principles will clash. The conflict is exacerbated by the fact that the explantation of an implant is both an intervention (as is all surgery) and the discontinuation of an intervention. *Qua* intervention it is disallowed since it harms the patient, but *qua* discontinuation it is required since the patient has a right to end her treatment. In the most extreme cases, such as a wish to have a total artificial heart removed or deactivated, complying with a patient’s request would be tantamount to killing her [26].

Two recent cases from the literature illustrate the dilemma. In one case, a patient with a unilateral cochlear implant asked to have it removed in order to strengthen her identity as a member of the Deaf culture. She was offered deactivation, or partial removal that would allow for later reimplantation, but neither of these alternatives would satisfy her. She was informed that the explantation she required would make reimplantation impossible, and that a later implantation in her non-implanted ear would be impossible due to ossification. In spite of this, she did not change her mind. After extensive consultations, the implant was removed [27]. The explantation surgery was fairly uncomplicated. The decision might have been different for an explantation that could pose larger risks of complications, such as the removal of a brainstem implant or vestibular implant.

The other case was a patient who asked to have his implantable cardioverter defibrillator (ICD) removed, since it was aesthetically unattractive and he did not believe he needed it. Many attempts were made to convince him that it had already saved his life twice and that without it, he was likely to die soon due to a cardiac arrest. Nevertheless, he insisted to have it explanted, and after extensive consultations, it was removed. Four months later, he had a cardiac arrest. He was lucky to have a person close by who could offer immediate assistance, and he was brought to an intensive care unit where his life was saved. He then demanded and received a new ICD [28].

No simple solution to the ethical dilemma illustrated in these examples seems to be available. The principles of autonomy and non-maleficence have to be weighed against each other, and the outcome of that weighing will be different depending on what is at stake in each individual case. However, it should be observed that in cases when an explantation would predictably lead to the patient’s death, the intervention is highly problematic even with the patient’s consent [29, 30]. In many countries it can be a criminal offence. The vast majority of patients whose life could by ended by explantation or some other intervention by a physician are in a position in which they could have ended their lives by their own means. A wish that a physician ends one’s life should be seen in that perspective [31, 32]. An adequate medical response to a wish to commit suicide should include a psychiatric assessment and, if indicated, an offer of psychiatric treatment. Consultation with a clinical ethicist is also highly advisable in this type of cases.

Although this dilemma has no general solution, measures can be taken to reduce the number of cases in which it has to be dealt with. Patients offered an implantation should receive adequate information about potential future interventions, including explantation. If it can be foreseen that explantation would be considered a breach of professional ethics, and would therefore be denied, then the patient should be informed of this. As noted by Owoc and coworkers, there is a need for explantation guidelines to “assist in clinical decision-making and patient counselling and education” [27]. Even with such guidelines, consultations with relevant specialties, including clinical ethics, will be needed before decisions are made on elective explantations that are medically contraindicated.

### Ending clinical trials

Clinical trials with implants give rise to two complex issues involving explantation, namely (1) how to deal with a participant’s wish to leave the trial and have the implant removed, and (2) whether it is acceptable to remove an implant after the trial if the patient wants to keep it.

One of the generally accepted principles of research ethics is that participants in a clinical trial have the right to withdraw their informed consent at any time, and that the withdrawal should be respected and implemented by the researchers [33]. Just as a patient with decisional capacity has the right to end a clinical treatment, a subject in a trial has the right to end the experimental treatment. In a recent article, a group of clinicians and ethicists recognize that research participants “arguably have a right of self-determination to refuse the continued presence of an invasive device in their bodies”. However, they maintain that this must be weighed against “the feasibility of imposing additional burdens on the research enterprise”. They conclude that “researchers should not be obligated to cover costs related to device removal if it is incompatible with the sustainability of the research enterprise that initiates the relationship that grounds the obligation in the first place” [34]. This is a remarkable argument, since it makes the basic rights of research subjects in relation to researchers secondary to the economic viability of the research project. A clinical trial should not be performed if lack of economic resources would prevent participants from exercising the rights, including the right to leave the trial at any time, that are standardly required and implemented in clinical trials.

But there is an exception, namely the comparatively unusual case of trials of a life-sustaining technology. One example is trials of total artificial hearts. A request to withdraw from such a trial and have the device explanted would be equivalent to a request to have one’s life ended. This is essentially the same situation as when a patient requires the removal of a life-sustaining device that was implanted in a non-experimental setting. The above considerations of the latter case apply in a clinical trial as well. Current regulations and ethical principles that stipulate an exceptionless right to leave a trial do not seem to have taken this unusual case into account [35]. (However, clauses concerning explantation and deactivation are included in a published consent form for a trial with total artificial hearts [36].)

For some participants, a clinical trial can be a short period of improved health, followed by deterioration when the experimental treatment ends. This has been the case in some drug trials, such as trials of HIV/AIDS treatments in developing countries. It now seems to be generally agreed that this is an unacceptable practice, and that funders and organizers of clinical trials have to make sure that participants receive adequate post-trial treatment [37]. For implant trials the situation can be even more drastic, since terminating the treatment at the end of the trial will typically require either an explantation or abandoning a non-functional device in the body, both of which can involve risks.

One problematic example is the Dobelle project, which started around 1975 and ended in 2005 [38]. The subjects were blind people who had electrodes implanted on the surface of the visual cortex. These electrodes were connected to converted camera images that gave rise to phosphene patterns. Some of the subjects were able to use these patterns for orientation or object detection. They felt a profound loss when they were deprived of these abilities due to device failure or the termination of the study. (This study also had other questionable features from an ethical point of view. Subjects had to pay up to 200,000 USD for participation, and although the project was American, the implantations took place in Portugal due to lack of FDA approval.)

A recent article described a trial with deep brain stimulation against treatment-resistant depression. Some participants who had experienced an improvement with the implant wanted to retain it after the end of the trial, but this was only possible if they could pay for it themselves or some charitable donor could be found. The research project only covered the costs of explantation or a rechargeable battery. According to the authors, “[t]his is the norm, not the exception, in brain-implant trials. In fact, most sponsors do not cover the cost of device removal or a rechargeable battery” [39]. Thus, these trials have the withdrawal of beneficial treatments that patients desire as a planned and foreseen consequence. This does not seem to be compatible with the CIOMS/WHO guidelines, according to which researchers and sponsors of trials have to make plans for “providing continued access to study interventions that have demonstrated significant benefit” [40]. Joseph Fins has proposed that the neuromodulation community should adopt an ethical principle of non-abandonment. “After a subject is implanted” in a trial, he says, the investigors and sponsors “incur a clinical responsibility to provide on-going care and a fiscal responsibility for any associated costs. It is a breach of professional ethics to do otherwise” [41]. The same principle can be applied to other implants. An experimental implant can be explanted because the patient does not need or want it any more, but it is not ethically acceptable to explant it because the patient cannot pay for continued clinical use. A clinical trial with implants should not be performed if it would result in patients being abandoned, or forced to have functional implants explanted, for economic reasons. Performing such studies would be at variance with the Helsinki declaration, which requires provisions to be made for “post-trial access for all participants who still need an intervention identified as beneficial in the trial”. It would also endanger public trust in medical research [42].

### Explanted implants

Just as removed organs or parts of organs are sent to pathology, investigations of explanted implants can provide useful information both in the individual case and for general medical and technological improvement. This applies in particular to failed implants, but also to devices that are explanted for other reasons than technical failure. The evaluation of an explanted device should be made on the basis of relevant clinical information about its functioning and the patient’s experiences [43]. Although detailed methods for the analysis of retrieved implants have been developed, such analyses are still not performed in the systematic way that removed tissues are sent to pathology [44–46].

Explanted but still functional implants can in some cases be reused [47]. In several industrialized countries, removed pacemakers with sufficient battery capacity are collected for use in low- and middle-income countries. Some of these pacemakers are explanted in the clinic, whereas others are removed post-mortem, which is necessary to avoid explosions in crematoria. After evaluation and sterilization, these pacemakers are sent to cardiology clinics in low- and middle-income countries [48]. The need for pacemakers is large; one charity organization estimates that about one million people a year die for lack of a pacemaker [49]. The ownership of used pacemakers has been cited as a problem that can prevent the reutilization of pacemakers. In the 1990s, when explanted functional pacemakers were reused domestically in industrialized countries, the legal situation shifted between countries. In Sweden, the implanting medical centre was considered to own the device, whereas in Canada and the Netherlands it was considered to be the property of the patient, or, after death, the patient’s heirs [50, 51]. Today, it is generally assumed that informed consent from the patient or family is required, and consent forms are collected for all pacemakers [48].

## Discussion

Several of the ethical issues identified above concern the control and decision-making power over implants. Does the patient have a right to have an implant removed, even if the explantation is medically harmful? Does a participant in an implant trial have the right to have the implant explanted if she wishes to leave the trial? Does she have the right to keep the implant after the end of the trial? Who should decide on the fate of explanted implants, for instance whether they should be sent to technical investigation and/or be restored for reuse in another patient?

In the comparatively small literature in the appropriate legal status of implants, it has usually been recognized that patients should have a considerable degree of control over implants, not least since implants “play a role in constituting a person—enabling continued life and contributing to the person’s capacities to act and experience” [52]. However, control over implants has usually been conceived in terms of ownership, and some discussants have seen the recognition of the patient’s ownership of (in situ or explanted) implants as the best way to guarantee patient autonomy [43]. This line of thought takes it for granted that the legal construct of ownership, which serves us well in protecting our rights to various goods that we have acquired lawfully, is also the right legal construct for patient rights concerning implanted medical devices. However, ownership is not the only way in which a person’s rights to an object can be regulated. Some of our most important rights are protected by other types of legal constructs. In a properly functioning democracy, your right to vote and your right to freedom are of a different nature. The most important difference is that whereas you can sell or give away objects that you own, your voting right and your freedom are inalienable. You can neither sell your vote to someone else, nor sell or give away yourself as a slave. Immanuel Kant pointed out that our control over our body parts is of a similar nature since “a man is not entitled to sell his limbs for money”. His main argument for this was that the body “constitutes a part of our self”, from which follows that “one cannot dispose over it, as though it were an end” [53].

For our present purposes, Kant’s justification is of less interest than his standpoint that a person’s right to her body parts is inalienable and therefore of an entirely different nature from ownership of a commodity. There are other ways to justify this distinction [54]. (A particularly interesting alternative is the privacy-based approach proposed by Rao [55].) As argued more extensively elsewhere, the notion of such an inalienable right is highly useful in explicating the rights a person has to her body and its parts [56]. It can also provide useful guidance for the rights that patients have to their implants. To make this more precise, it is proposed that *if an implant replaces a part of the body, or fills its function to a significant degree, then the person has essentially the same type of right to that implant as she has to her original, biological body parts*. The same applies to a transplanted organ. We can call transplants and implants that satisfy this criterion *supplemented body parts*. The rights that a person has to a supplemented body part derive from its function in her body, not from it being given or sold to her. This account provides rather immediate answers to important ethical questions concerning implants and their explantation:

- A patient has a strong right to retain an implanted device that fulfils or contributes to fulfilling a function in her body. This also applies if it was implanted in a clinical trial.
- If an implant loses its functionality, then it also loses its status as a supplemented body part. However, the usual conditions for medical interventions, including informed consent and non-maleficence, apply to its explantation.
- The criteria for when such an implant can legitimately be turned off are the same as the criteria for when corresponding actions can be applied to a biological organ. This speaks against turning off such implants on grounds of futility [57]. In particular, it speaks strongly against Katrina Bramstedt’s claim that turning off a total artificial heart on grounds of futility is ethically much the same as turning off dialysis or ventilation in a similar situation [26, 58].
- The patient’s strong rights to an implant vanishes when it has been explanted and no longer fills a function in her body. After explantation, the implant becomes an ordinary object that can be owned in the usual sense of the word. Patients’ rights are not violated if removed implants become the property of a healthcare organization. Neither are they violated by rules or practices that ensure the investigation of explanted implants for the purpose of medical and technological improvement [59], or by reuse of the implant in another patient who needs it. (It can reasonably be argued that a person’s inalienable right to a biological body part vanishes in the same way after it has lost its functionality and been removed from the body. However, this seems to be of less practical importance since there is not much use for removed dysfunctional organs.)

It remains to determine under what conditions an object inserted into a person’s body acquires the status of a supplemented body part. No complete treatment of that issue can be given here, but some indications can be given of what criteria should be decisive. Considerable weight should be assigned to the patients’ own perceptions of the implant. For instance, a left ventricle assist device is commonly perceived as part of one’s body, whereas an implantable cardioverter-defibrillator is more often perceived as an alien addition to it [32, 60]. This makes the former a stronger candidate than the latter for the status as a supplemented body part. (However, a person’s perception that a device is functionally a part of herself cannot be a sufficient condition for assigning to that device the same moral and legal status as a body part. This would lead to the absurd conclusion, supported by some authors, that a laptop or smartphone, can be given that status [61, 62]).

Another criterion that should be given considerable weight is that the device fulfils at least a significant part of the function of some original biological organ. What should count is the function fulfilled, not the physical way in which it is fulfilled. For instance, a “pulseless” artificial heart, based on a centrifugal pump, would be an equally strong candidate for the status as a supplemented body part as a pulsating artificial heart [30]. This can be further clarified with the help of an important insight from the philosophy of technology, namely that technological artefacts have a “dual nature”. They can be described either in terms of their function or in terms of their physical construction [63]. Discussions on whether an implanted medical device qualifies as a supplemented body part should be conducted in terms of functional, rather than physical, descriptions of the device.

## Conclusions

This investigation of the ethics of explantation has identified two major philosophical issues that both have implications for clinical ethics. First, an explantation is both an intervention (as surgery) and the end of an intervention (as removal of a therapeutic device). This gives rise to a moral dilemma if a patient requires an explantation that is deemed to be medically harmful. Non-maleficence forbids the explantation since it is a harmful intervention, whereas patient autonomy demands it since it is needed to end an intervention that the patient no longer desires.

Secondly, several important issues in explantation ethics depend on the control and decision-making power over the implant. We have proposed that like transplants, implants that fulfil functions normally carried out by biological organs can qualify for the status of supplemented body parts. This means that as long as the implant is functional, the patient has a strong and inalienable right to it. Such a right provides a better basis than the common legal construct of ownership for analyzing the ethical issues pertaining to implants and their explantation.

Our investigation has also resulted in a number of concrete proposals for ethical standpoints concerning explantation:

1. Pre-implantation information to patients should include information pertaining to a possible future explantation.
2. Decisions on whether or not to explant should be based on careful analysis of the potential risks and advantages to the individual patient. A manufacturer’s recall is not necessarily a sufficient reason to remove an implant.
3. Safe routines must be in place to ensure that patients with temporary implants are reached when it is time to have the implant removed.
4. Routines for investigations of explanted implants should be strengthened in order obtain useful information for medical and technological development.
5. Participants in implant trials have the right to leave the trial at any time, and then have the implant removed.
6. Participants in implant trials who want to keep the implant after the end of the trial due to its medical benefits should have the right to do so free of charge.
7. Additional registers of patients with implants should be introduced in order to facilitate epidemiological studies and make it possible to reach patients with potentially defective implants.
8. The quality assurance system for implants needs to be improved. Like the system for pharmaceutical drugs, it should include both pre-market clinical trials and post-market studies. Full transparency is needed to ensure that independent researchers can analyze and compare the data for different implants.
9. Manufacturers should market fewer but more thoroughly tested models of implants.
10. Manufacturers should give higher priority to increased service time in battery-driven implants.

## Data Availability

Not applicable.
